# Antifungal activity and genomic characterization of the biocontrol agent *Bacillus velezensis* CMRP 4489

**DOI:** 10.1038/s41598-022-22380-0

**Published:** 2022-10-18

**Authors:** Julia Pezarini Baptista, Gustavo Manoel Teixeira, Maria Luiza Abreu de Jesus, Rosiana Bertê, Allan Higashi, Mirela Mosela, Daniel Vieira da Silva, João Paulo de Oliveira, Danilo Sipoli Sanches, Jacques Duílio Brancher, Maria Isabel Balbi-Peña, Ulisses de Padua Pereira, Admilton Gonçalves de Oliveira

**Affiliations:** 1 Department of Microbiology, Universidade Estadual de Londrina, Londrina, PR 86057-970 Brazil; 2 Universidade Tecnológica Federal Do Paraná, Cornélio Procópio, PR 86300000 Brazil; 3 Department of Computer Science, Universidade Estadual de Londrina, Londrina, PR 86057-970 Brazil; 4 Department of Agronomy, Universidade Estadual de Londrina, Londrina, PR 86057-970 Brazil; 5 Department of Preventive Veterinary Medicine, Universidade Estadual de Londrina, Londrina, PR 86057-970 Brazil; 6 Laboratory of Electron Microscopy and Microanalysis, Universidade Estadual de Londrina, Londrina, PR 86057-970 Brazil

**Keywords:** Biotechnology, Computational biology and bioinformatics, Applied microbiology

## Abstract

The development of bio-based products has increased in recent years, and species of the *Bacillus* genus have been widely used for product development due to their elevated production of antimicrobial molecules and resistance to extreme environmental conditions through endospore formation. In this context, the antifungal potential of *Bacillus velezensis* CMRP 4489 was investigated using in silico predictions of secondary metabolites in its genome and in vitro tests against the following phytopathogenic fungi: *Sclerotinia sclerotiorum*, *Macrophomina phaseolina*, and *Botrytis cinerea*. The in-silico predictions indicated that CMRP 4489 possesses several Biosynthetic Gene Clusters (BGCs) capable of producing molecules with antifungal properties and other non-identified BGCs. The in vitro assay results evidenced strong antifungal activity, inhibiting more than 60% of the tested fungi, and the isolate’s molecules were stable under diverse physicochemical conditions. The in vitro assay evidenced significant antifungal activity, deformation of the hyphal structure in SS, biofilm formation capacity, and swarming motility. In the colonization assay, we observed attachment, colonization, and net-shaped biofilm formation, with the strain transitioning from the seeds to nearby structures. Therefore, CMRP 4489 showed to be a potential biocontrol agent against various diseases with agronomic importance and can be used under adverse environmental conditions.

## Introduction

The world agrarian sector currently recognizes environmental issues as a positive agenda in favor of more efficient, higher-quality production, capable of limiting the expansion of agricultural frontiers and environmental toxicity^1^. Producers, who in recent decades have invested in the development of specialized and highly technological agriculture for greater productivity, are also considering the recovery of degraded areas, investments in the diversification of their properties, crop rotation, mixed planting, and the use of biological control agents (BCAs)^2^.

The use of BCAs as a form of management or integrated management to protect crops against pests, weeds, and microbial diseases for more sustainable food production and to cause less impact on the environment corroborates the aspirations of a society that increasingly demands safer and cleaner products and processes that are more ecofriendly^3^. In addition to environmental and public health factors, it is important to highlight the emergence of a new economic trend—bioeconomy—focused on bio-based industries and businesses^4^.

BCAs are widely used in many fields, including agriculture, medicine, and forestry products. Multiple genera have been studied as potential biocontrol agents. However, the *Bacillus* genus deserves mention because of its high capacity to synthesize a vast array of beneficial substances of agronomical and industrial interest, as well as being highly resistant to elevated temperatures due to its ability to form endospores^5,6^, thus being used in scale in bio-based industries.

Among the different species of the genus *Bacillus*, *B*. *velezensis* stands out due to its significant environmental versatility and for producing numerous antifungal compounds, such as lipopeptides^7^, enzymes^8^, and volatile compounds^9^, in addition to acting as a Plant Growth-Promoting Rhizobacteria (PGPR)^10^. Therefore, evaluating a new strain of *B. velezensis* could lead to the discovery of novel bioactive compounds, as well as position this species as a promising biocontrol agent for the bio-industry. In the present study, we aimed to assess the antifungal activity and stability of the extracellular metabolites of *B. velezensis* strain CMRP 4489, its influence in the suppression of *Sclerotinia sclerotiorum* on soybean seeds, and its biosynthetic potential, based on genome mining.

## Materials and methods

### Plant-pathogenic fungi

Fungal isolates of *S. sclerotiorum* (SS), *Macrophomina phaseolina* (MP), and *Botrytis cinerea* (BC) were grown in potato dextrose agar (PDA) medium (Neogen Corporation, USA) at 25 °C, with a photoperiod of 12 h/12 h, and stored in the same medium at 4 °C. All isolates were deposited in the Microbial Culture Collection of the Plant Pathology Laboratory of the Department of Agronomy of the State University of Londrina.

### Biological control agent

The *B. velezensis* strain CMRP 4489 (LABIM40) used in this study was originally isolated as an antagonistic contaminant on a plate with fungal growth at the State University of Londrina (UEL), in Paraná, Brazil. Its complete genome has been sequenced and announced^11^. CMRP 4489 was maintained at the Laboratory of Microbial Biotechnology at UEL and was also deposited as strain CMRP 4489 in the *Coleções Microbiológicas da Rede Paranaense* (CMRP) network of the Federal University of Paraná, in Curitiba, Brazil.

### Phenotypic characterization, colony architecture, and identification of B. velezensis CMRP 4489

*B. velezensis* CMRP 4489 was grown in Luria–Bertani (LB) broth medium (Neogen Corporation, USA) at 28 °C, and, after 24 h, Gram staining was performed using a Gram-staining kit for morphology and cell wall visualization. Endospore formation was observed using the Wirtz-Conklin method after 48 h of incubation. Scanning electron microscopy (SEM) was conducted to visualize colony morphology. In this step, a colony grown for 48 h was removed from the agar and fixed in a solution containing 2.5% glutaraldehyde and 2% paraformaldehyde in 0.1 M sodium cacodylate buffer (pH 7.2) at 4 °C. The samples were kept in this solution overnight for fixation, after which they were washed three times with 0.1 M sodium cacodylate buffer (pH 7.2) for 10 min, followed by dehydration three times for 10 min in an ethanol series (30, 50, 70, 90, and 100%). The samples were then submitted to critical point drying with CO_2_ (BALTEC CPD 030 Critical Point Drier), coated with gold (BALTEC SDC 050 Sputter Coater), and observed with an FEI Quanta 200 scanning electron microscope (SEM) operating at 25.0 kV. For strain identification, ANI (Average Nucleotide Identity) and dDDH (digital DNA-DNA Hybridization) analyses were carried out using the Orthologous Average Nucleotide Identity Tool^12^ and the Genome-to-Genome Distance Calculator^13^. For the phylogenetic analysis, we selected the genomes of all strains classified as reference sequences of the genus *Bacillus* deposited in the GenBank database. Comparative data of the genomes were obtained using the Gegenees software^14^, which provides a data matrix on the phylogenetic relationships between strains. For the construction of the phylogenetic tree, the generated data were exported to the SplitsTree program^15^, where the tree was created using UPGMA (Unweighted Pair Group Method using Arithmetic averages), which calculated the evolutionary distance between the analyzed strains.

### Floating pellicle biofilm and swarm expansion assays

The CMRP 4489 strain was evaluated regarding floating pellicle biofilm formation in 24-well plates. An inoculum was prepared from a culture grown overnight at 28 °C in LB medium (Neogen Corporation, USA), and the suspension was adjusted according to the 0.5 McFarland scale, rendering approximately 1.5 × 10^8^ colony-forming units/mL (CFU/mL). Afterward, 10 µL of the inoculum were added to a 24-well plate (Sarstedt, USA) containing 2 mL of LB medium, which was incubated at 28 °C for 24 h. For the swarm expansion assays, the CMRP 4489 inoculum was prepared as described above. Afterward, 90 mm-diameter Petri dishes containing LB medium with 0.7% agar (Neogen Corporation, USA) were dried in a biological safety chamber (Filterflux, Brazil) for 30 min and inoculated centrally with 10 µL of the inoculum, dried for another 10 min, and incubated at 28 °C for 24 h.

### Antagonistic activity

Using an in vitro dual-culture assay, the CMRP 4489 inoculum was subjected to antagonism assays against SS, MP, and BC in PDA medium. To this end, 6 mm agar plugs containing 7-day-old fungal mycelia were placed at the center of 90 mm Petri dishes. Five microliters of the CMRP 4489 inoculum (prepared as described in Subheading—*Floating pellicle biofilm and swarm expansion assays*) were transferred onto the Petri dishes at approximately 25 mm from the fungal mycelia plugs at four equidistant points. The plates were incubated at 25 °C with a photoperiod of 12 h/12 h. Antifungal activity was expressed as the percentage of mycelial growth inhibition (MGI), according to the following formula:

$$ MGI \left( \% \right) = \left[ {\frac{{\left( {C - T} \right)}}{C}} \right] \times 100 $$

where *MGI* (%) is the percentage of mycelial growth inhibition; *C* represents the colony radius of the fungal control plates, and *T* is the radius of the fungal colony in the treatment plates^16^. The experiment was repeated twice with 8 replicates, and the results were submitted to analysis of variance (ANOVA) and the means compared by the Tukey test (*p* < 0.05).

### Antifungal metabolite production

The CMRP 4489 strain was activated on Luria–Bertani agar (LBA) (Neogen Corporation, USA) and incubated at 28 °C for 24 h. For the preparation of the pre-inoculum, colonies were suspended in saline solution (0.85% sodium chloride, w/v), and the concentration was adjusted according to the 0.5 McFarland scale until reaching approximately 1.5 × 10^8^ CFU/mL. For the preparation of the inoculum, 30 µL of pre-inoculum were inoculated separately in 125 mL Erlenmeyer flasks containing 30 mL of two culture media (CM1 or CM2) and incubated at 28 °C for 24 h at 125 rpm (Orbital shaker—Thoth 6430B, Brazil). Afterward, a 1% aliquot of the final volume (v/v) was transferred to a 1000 mL Erlenmeyer flask containing 400 mL of CM1 or CM2:

CM1–g/L: tryptone 10.0; yeast extract 5.0; NaCl 5.0; pH 7.1.

CM2–g/L: glucose 20.0; tryptone 12.4; NaCl 5.0; K_2_HPO_4_·3H_2_O 1.5; MnSO_4_·H_2_O, 0.04; FeSO_4_·7H_2_O, 1.67; MgCl_2_ · 6H_2_O, 1.22; pH 7.1.

All culture media were incubated at 28 °C for 72 h at 150 rpm (Orbital shaker—Thoth 6430B, Brazil). Next, the fermentations were centrifuged for 10 min at 8860 × *g* at 4 °C (Hitachi, CR21G Himac, Japan) to obtain the cell-free supernatants (CFS). All CFS were sterilized by filtration through a cellulose filter with a pore size of 0.22 μm (Millipore, USA), thus obtaining CFS-CM1 and CFS-CM2.

### Antifungal activity of the cell-free supernatants

CFS-CM1 and CFS-CM2 were checked for antifungal activity using the agar well diffusion method against SS, MP, and BC (prepared as described in Subheading—*Antagonistic activity*). For each fungus, 6 mm agar plugs containing 7-day-old fungal mycelia were placed at the center of 90 mm Petri dishes. Four symmetrically distributed wells were made approximately 25 mm from the fungal mycelia plugs. A total of 200 μL of CFS-CM1 and CFS-CM2 were added to the wells, separately per Petri dish (4 experimental units with 4 wells). Sterile CM1 and CM2 were used as blanks. The plates were incubated at 25 °C with a photoperiod of 12/12 h for 96 h. The experiment was repeated twice. The results were submitted to analysis of variance (ANOVA) and the means compared by the Tukey test (*p* < 0.05). Scanning electron microscopy was performed to visualize the antifungal activity of the cell-free supernatants (CFS) of the best culture medium identified.

### Ultrastructural damage evaluated by scanning electron microscopy

SEM was carried out to visualize the ultrastructural damage against SS of the CFS obtained from the best culture medium identified. In order to observe the ultrastructural damage, an evaluation was carried out at the inhibition zone-SS mycelial growth interface. The control was treated with sterile culture medium, and the assay was conducted in accordance with the method described previously. Plugs measuring 6 mm in diameter were cut and placed in vials containing 2.5% glutaraldehyde and 2% paraformaldehyde in 0.1 M sodium cacodylate buffer (pH 7.2) at 4 °C. The samples were kept in this solution overnight for fixation, after which they were washed three times with 0.1 M sodium cacodylate buffer (pH 7.2) for 10 min. Subsequently, the samples were dehydrated three times in an ethanol series (30, 50, 70, 90, and 100%) for 10 min. The samples were submitted to critical point drying with CO_2_ (BALTEC CPD 030 Critical Point Drier), coated with gold (BALTEC SDC 050 Sputter Coater), and visualized with an FEI Quanta 200 scanning electron microscope operating at 25.0 kV.

### Growth curve and antifungal metabolite production at different incubation times

The inoculum preparation methodology used to produce antifungal metabolites and obtain the CFS was carried out as previously described using the best culture medium. The samples were removed at different time intervals (0, 12, 24, 36, 48, 60, and 72 h) to determine antifungal activity and bacterial growth. The antifungal activity of the CFS was assessed using the method described previously. Bacterial growth was evaluated according to cell count (CFU/mL) from tenfold serial dilutions in sterilized saline solution (0.85% sodium chloride, w/v) plated on MYP agar (Neogen Corporation, USA) and incubated at 28 °C for 24 h. This assay was repeated twice. Pearson’s correlation coefficient was used to investigate the correlations between growth and antifungal activity. Statistical significance was considered when *p* < 0.05.

### Evaluation of stability of the antifungal metabolites at different temperatures, pH, and light wavelengths

In order to evaluate the stability of the antifungal metabolites at different temperatures, pH, and light wavelengths, aliquots of CFS from the best culture medium were used. The samples were exposed to constant temperatures of 28 °C, 70 °C, and 100 °C for 30 min, and 121 °C for 15 min. Subsequently, the samples treated at 28 °C, 70 °C, and 100 °C were sterilized by filtration through a cellulose filter with a pore size of 0.22 μm (Millipore, USA), and antifungal activity was assessed according to the method described previously. The experiment was performed three times, with eight repetitions each, and linear regression analysis (*p* < 0.05) was conducted to represent the data. For the pH stability assay, the samples were adjusted to various pH values in the range from 3.0 to 11.0 using 2.0 M HCl or 2.0 M NaOH and maintained at 4 °C for 24 h. Afterward, the samples were readjusted to pH 7.0^17^, sterilized by filtration through a 0.22 μm cellulose filter (Millipore, USA), and submitted to the antifungal activity assay described previously. The evaluation was carried out in two independent experiments, with 4 replicates each, and was based on descriptive analysis. As for the determination of the effect of ultraviolet radiation (365 nm) and white light, the samples were exposed to the two for 12 h at a distance of 15 cm. The untreated CFS was used as a control. After that, all samples were sterilized by filtration through a 0.22 μm cellulose filter (Millipore, Bedford, MA), and their antifungal activity was assessed according to the method described previously. Six repetitions were performed in two independent experiments. The assay was later evaluated using the Kruskal–Wallis nonparametric test (*p* < 0.05).

### B. velezensis CMRP 4489 inoculation on soybean seeds: colonization assays by SEM

Seeds of the soybean cultivar 6461RSF IPRO were disinfected with 2% sodium hypochlorite for 2 min and then washed 3 times with sterile water and dried in a biological safety chamber for 20 min. After disinfection, one hundred soybean seeds were treated, in 125 mL Erlenmeyer flasks, with strain CMRP 4489 cultivated in CM2 and adjusted to 5 × 10^9^ CFU/mL (dose of 200 mL/100 kg). The control seeds were treated with CM2 (200 mL/100 kg) alone. After manually homogenizing the flasks for 2 min, 10 seeds were transferred to a 150 mm Petri dish containing a filter paper moistened with sterile deionized water. In order to evaluate cell adhesion on day 0 and compare it with the uninoculated seeds, 3 of both inoculated and uninoculated seeds were fixed for further evaluation by SEM. The remaining seeds were incubated in a humid chamber for 7 days at 23 °C with a photoperiod of 12/12 h and, every 24 h, moistened with approximately 5 mL of sterile deionized water. After incubation, the tegument and radicle of 3 seven-day-old germinated seeds were fixed for SEM. The procedures used in the SEM assay were conducted according to the method described previously. The concentration of CMRP 4489 present in the seeds after treatment and the seeds and radicle after incubation were quantified^18^. Immediately after treatment, 10 seeds were added to 10 mL of saline solution and vortexed for 1 min for cell recovery. After 7 days of incubation, 10 radicles were incubated in 125 mL Erlenmeyer flasks with 10 mL of saline solution containing glass beads for 60 min, with agitation at 250 rpm at room temperature. The seeds corresponding to these rootlets were macerated using a mortar and a pestle, then added to 10 mL of saline solution. From the prepared extracts, the concentration of CFU/mL in the extract was determined by plating dilutions on MYP agar (Neogen Corporation, USA). The experiment was repeated three times in triplicate, and the initial and final total CFU/mL were compared by one-sided Student’s t-test (*p* < 0.05). The experimental procedures complied with institutional, national and international guidelines and legislations.

### B. velezensis CMRP 4489 inoculation on soybean seeds: suppression of Sclerotinia sclerotiorum

Seeds of the soybean cultivar Monsoy 6410 were disinfected according to the method described previously. After disinfection, five hundred soybean seeds were inoculated with *Sclerotinia sclerotiorum* (SS) for each treatment. To this end, SS was cultivated in PDA culture medium and, after 5 days of growth (100% of growth on 150 mm Petri dishes), the seeds were distributed onto the Petri dishes, maintaining contact with the fungal culture for 24 h. The treatments were as follows: (T1) control, with five hundred uninfected and untreated soybean seeds; (T2) control, with five hundred soybean seeds infected with SS without treatment; (T3) control, with five hundred soybean seeds treated with sterile CM2 (200 mL/100 kg); (T4) chemical treatment, with five hundred soybean seeds treated with a commercial product composed of 52.50 g/L fluazinam + 350.00 g/L thiophanate-methyl (200 mL/100 kg); (T5) biocontrol agent, with five hundred soybean seeds treated with strain CMRP 4489 cultivated in CM2 and adjusted to 5 × 10^9^ CFU/mL (200 mL/100 kg). All treatments were carried out in plastic bags and homogenized by manual shaking for 2 min. A layer of sterile filter paper was placed within 150 mm Petri dishes and moistened with sterile distilled water. Subsequently, 13 Petri dishes per treatment/control received 25 seeds and were incubated in a humid chamber at 23 °C, with a photoperiod of 12/12 h. The percentage of germinated seeds and uninfected seeds (absence of SS growth) was evaluated after 7 days of incubation. For the statistical analysis, the data were subjected to analysis of variance (ANOVA), considering a generalized linear model with binomial distribution. When significant, the treatments were compared using the Tukey test (*p* < 0.05). All analyses were processed using the R studio (v4.1.2). The experimental procedures complied with institutional, national and international guidelines and legislations.

### Genome mining: prediction of biosynthetic gene clusters and comparative analysis of genes involved in biofilm formation

The prediction of secondary metabolite clusters in the *B. velezensis* CMRP 4489 genome was carried out using the antiSMASH 6.0 webserver^19^. For the comparative analysis of genes involved in biofilm formation and swarming, a database was created using gene sequences associated with swarming and biofilm formation/regulation^20^, which were selected using data collected on the Subtwiki database^21^ and from studies related to root colonization^22–27^ by *Bacillus subtilis*. This developed database was used to compare similar sequences to the CMRP 4489 genome using the BLASTN program.

## Results

### Phenotypic characterization and colony architecture of B. velezensis CMRP 4489

Gram staining showed that CMRP 4489 is a Gram-positive, rod-shaped bacterium that produces endospores. Its rod-shaped morphology and cell association were evidenced by SEM (Supplementary Fig. 1). The analysis of dDDH and ANI (Supplementary Table 1) and the arrangement of the phylogenetic tree (Supplementary Fig. 2) indicate that strain CMRP 4489 belongs to the species *Bacillus velezensis*.

### Floating pellicle biofilm and swarm expansion

The in vitro assays evidenced the filming and swarming capacity of the CMRP 4489 strain. Microplate assays revealed a thick film over the entire air–liquid surface, formed after cell growth. In the swarming motility test, growth over the entire surface of the Petri dish was observed after overnight culture. These results indicate that this strain is capable of rhizospheric colonization, an important factor for the establishment of the plant-bacteria interaction (Supplementary Fig. 2).

### Dual-culture assay

**Figure 1:**
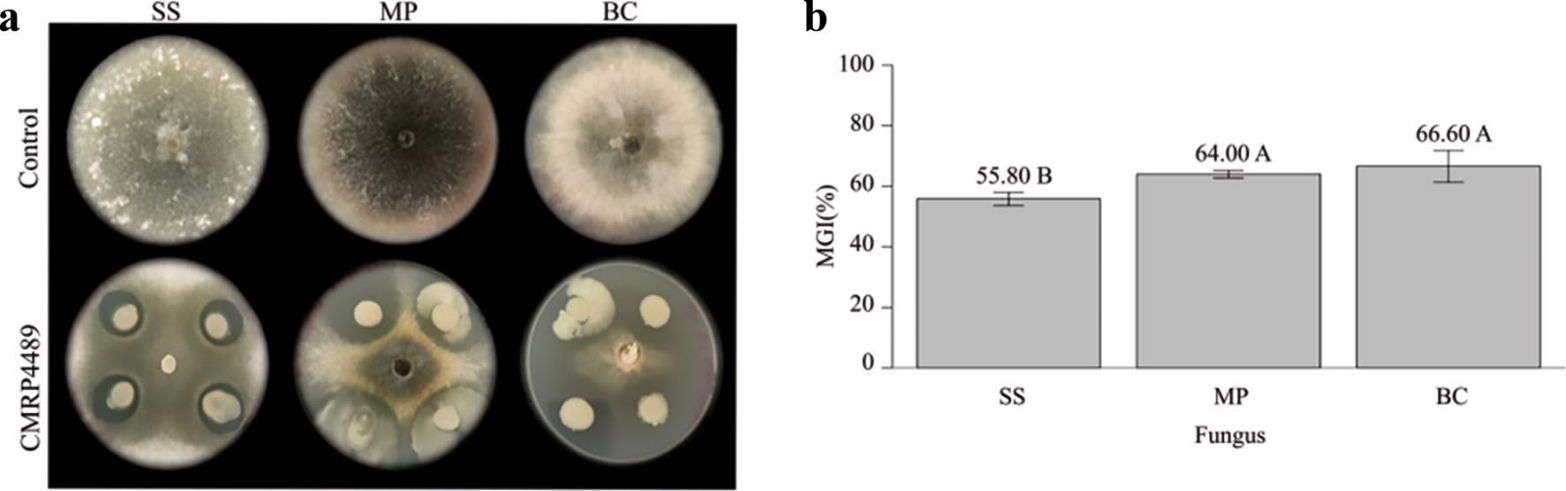
Dual-culture assay of *B. velezensis* CMRP 4489 and *Sclerotinia sclerotiorum* (SS), *Macrophomina phaseolina* (MP), and *Botrytis cinerea* (BC). (**a**) Photograph of the dual-culture test with the experimental controls. (**b**) Mycelial growth inhibition percentage (MGI%). Means followed by the same letter did not differ according to the Tukey test (*p* < 0.05).

The CMRP 4489 strain showed excellent antagonistic activity against SS, MP, and BC. In the experimental group, CMRP 4489 formed a very clear inhibition zone between the bacterial suspension and the pathogenic fungi (Fig. 1a). The mean inhibitory zone was measured, and the percentage of mycelial growth inhibition (MGI%) was calculated. The CMRP 4489 strain inhibited all the fungi, with 55.8%, 64.0%, and 66.6% inhibition of SS, MP, and BC, respectively (Fig. 1b).

### Antifungal activity of the cell-free supernatant

**Figure 2:**
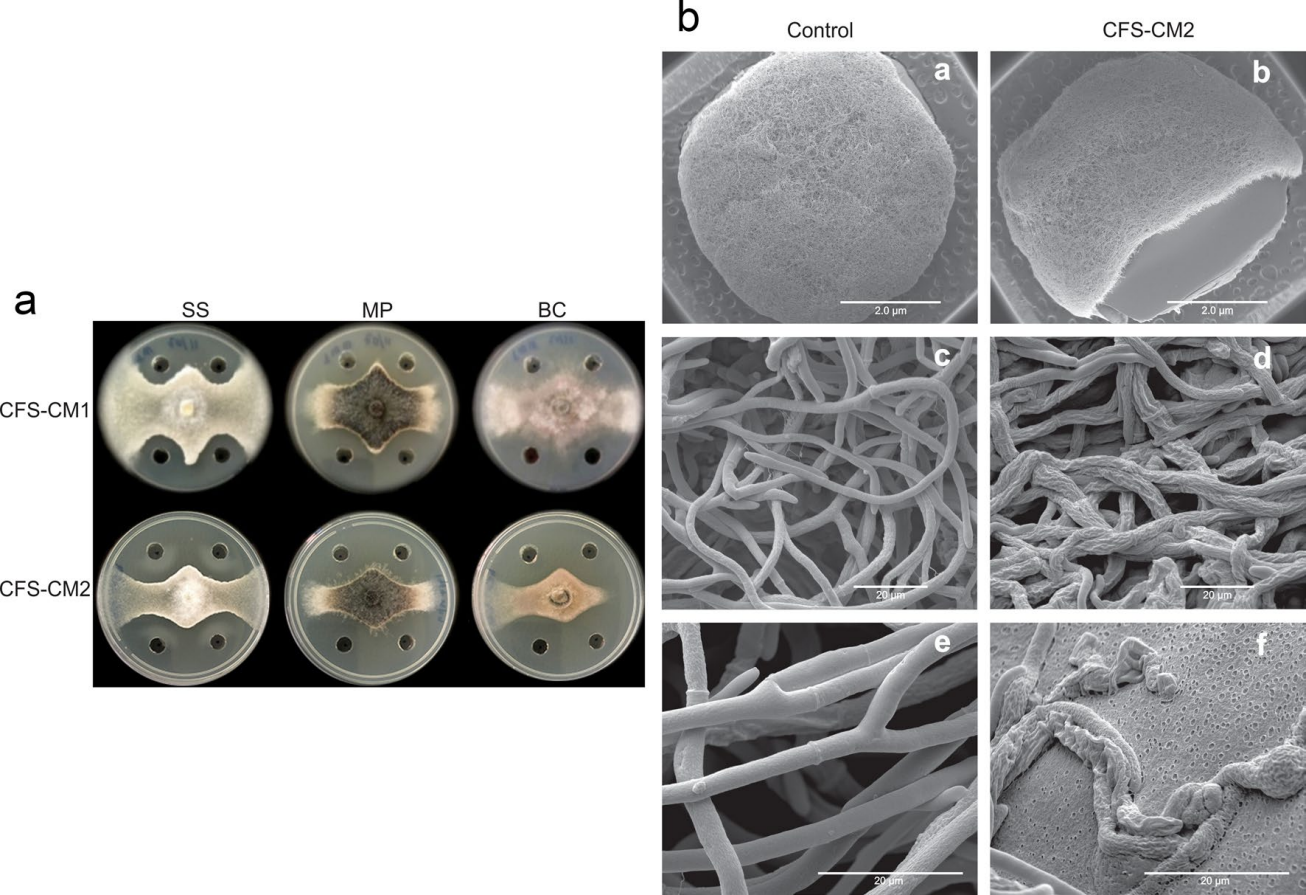
Antifungal effect of the cell-free supernatant (CFS) of *Bacillus velezensis* CMRP 4489. (**a**) Evaluation of antifungal activity against *Sclerotinia sclerotiorum* (SS), *Macrophomina phaseolina* (MP), and *Botrytis cinerea* (BC) by metabolites produced in CM1 and CM2 media. (**b**) Scanning electron microscopy images of the antifungal effect of the CFS against *Sclerotinia sclerotiorum*. The control refers to the untreated fungal spot and CFS-CM2 refers to the transition area between fungal growth and the inhibition region.

CFS-CM1 and CFS-CM2 showed excellent antifungal activity against the pathogenic fungi (Fig. 2a). When analyzing the effect of CFS-CM1 and CFS-CM2, it was observed that CFS-CM1 exhibited less antifungal activity, with a significant difference compared to CFS-CM2 (Table 1). The inhibitory values presented by CFS-CM1 reached up to 54.36% for SS, 57.27% for MP, and 36.76% for BC, with significant differences when comparing the three fungi. As for CFS-CM2, the inhibitory values reached 65.31% for SS, 68.31% for MP, and 72.87% for BC, with significant differences when comparing the three fungi (Table 1).

**Table 1:** Antifungal activity against *Sclerotinia sclerotiorum* (SS), *Macrophomina phaseolina* (MP), and *Botrytis cinerea* (BC) of the cell-free supernatants (CFS) obtained by submerged liquid fermentation of *Bacillus velezensis* CMRP 4489 in two culture media (CM1 and CM2).

Plant-pathogenic fungi	MGI (%)			
	CFS-CM1		CFS-CM2	
*Sclerotinia sclerotiorum* (SS)	54.36 ± 2.03	bA	65.31 ± 0.39	cB
*Macrophomina phaseolina* (MP)	57.27 ± 2.23	aA	68.31 ± 2.71	bB
*Botrytis cinerea* (BC)	36.76 ± 4.05	cA	72.87 ± 2.61	aB

MGI (%) ± Standard Error. Numbers followed by the same lowercase letter in the column (*p* < 0.05) and uppercase letter in the row (*p* < 0.01) did not differ according to Tukey’s test.

### Ultrastructural damage of Sclerotinia sclerotiorum

Considering that the CM2 medium showed better performance in producing the antifungal metabolites of the CMRP 4489 strain, it was selected for all of the following assays. The effect of CFS-CM2 on SS growth was substantial. The mycelia of SS in the control presented hyphae with a typical “net” structure and a smooth surface. In the presence of CFS-CM2, the hyphae lost their smoothness and formed unusual surface bulges, indicating that CFS-CM2 inhibited SS growth by causing deformation of the hyphal structure. The obtained results showed that CFS-CM2 distorted and damaged SS hyphae and that fungal growth was inhibited (Fig. 2b).

### Growth curve of the CMRP 4489 strain and the production of antifungal metabolites in CM2

**Figure 3:**
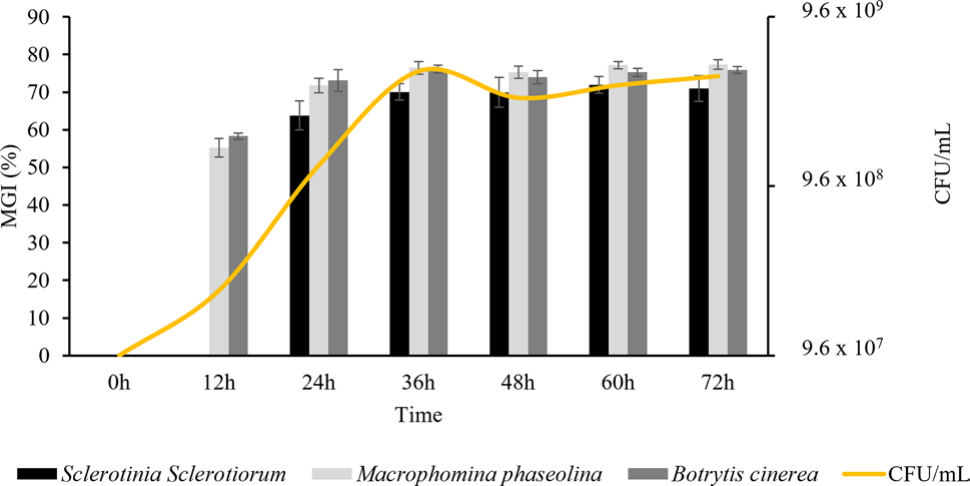
Kinetics of cellular growth and antifungal activity of the cell-free supernatant (CFS) of *Bacillus velezensis* CMRP 4489. Mycelial Growth Inhibition percentage (MGI%); Colony Forming Units per milliliter (CFU/mL).

The kinetic data on cell growth and antifungal activity are shown in Fig. 3. The culture of the *B. velezensis* CMRP 4489 strain was carried out at 28 °C in CM2 medium for 72 h. Accordingly, two phases could be distinguished during the fermentation process: a growth phase, until 36 h of incubation, followed by a stationary phase, which did not decline until 72 h of cultivation. No lag phase was observed. The production of the antifungal metabolites was dependent on the bacterial growth phase. The CMRP 4489 strain started producing antifungal metabolites at 12 h for BC and MP during the logarithmic growth phase. The strongest antifungal activity, with approximately 70% of inhibition of all fungi and 7 × 10^9^ CFU mL^-1^ of viable cells, was obtained at 36 h after incubation, which corresponded to the stationary growth phase, and did not decline over the next 36 h. Therefore, under the culture conditions used in this study, the optimal production time of antifungal metabolites for strain CMRP 4489 was 36 h after inoculation, when the viable bacterial biomass was the highest (Fig. 3).

### Effect of temperature, pH, and light on the CMRP 4489 supernatant

**Figure 4:**
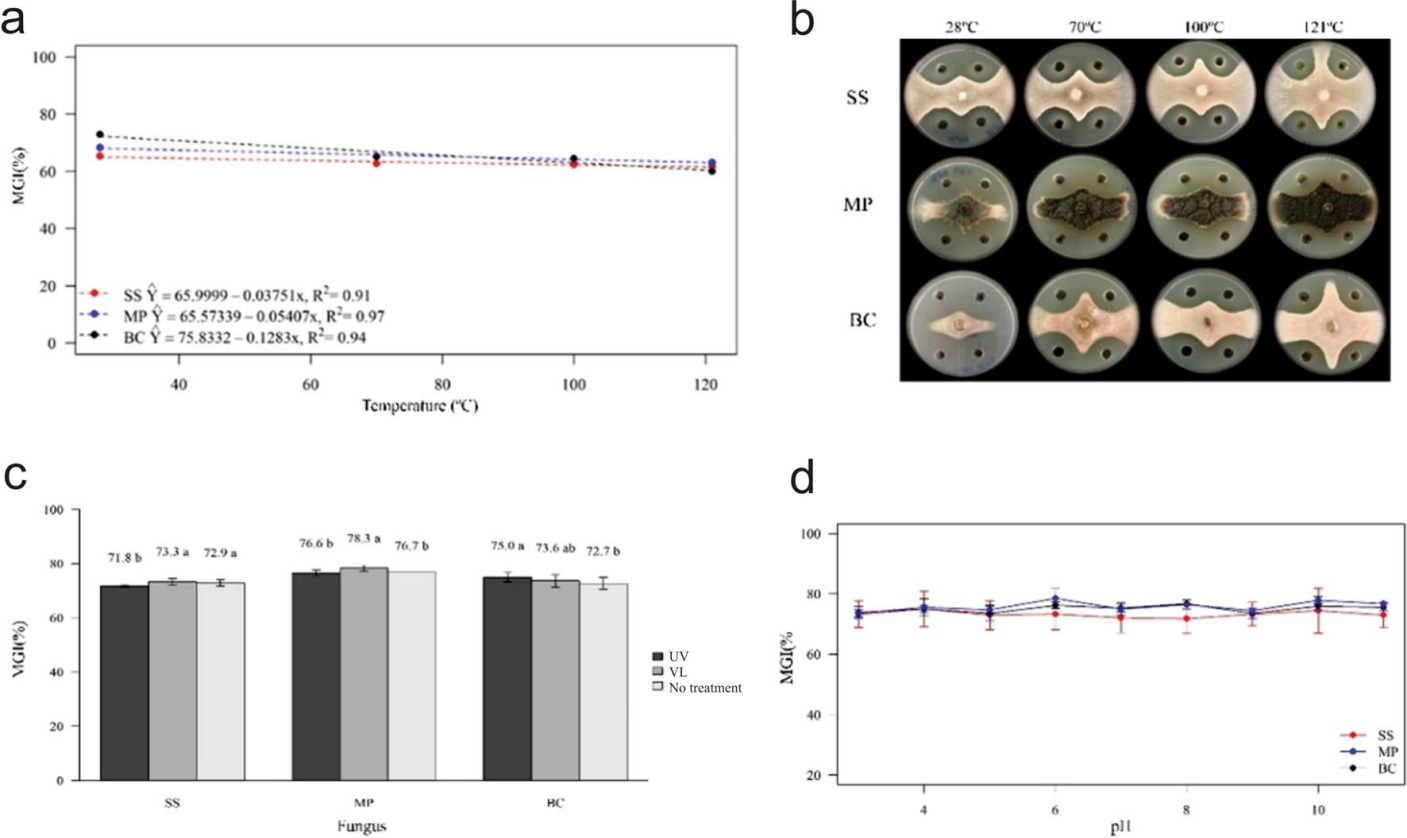
Mycelial growth inhibition (MGI%) of *Sclerotinia sclerotiorum* (SS), *Macrophomina phaseolina* (MP), and *Botrytis cinerea* (BC) by the cell-free supernatant (CFS) of *Bacillus velezensis* CMRP4489 after thermal, light, and pH treatments. (**a**) CFS stability at 28 °C (no treatment), 70 °C, 100 °C, and 121 °C. (**b**) Mycelial growth of SS, MP, and BC at different temperatures in the thermostability test. (**c**) CFS photostability assay in UV light (UV), at a wavelength of 365 nm, and visible light (VL). Same letters did not differ according to the Kruskal–Wallis test (*p* < 0.05). (**d**) CFS stability at different levels of pH (3–11).

The supernatant continued active after being subjected to high temperatures. However, its activity was lower after autoclaving, being more evident against BC, with an MGI of 73, 65, 64, and 60%; SS, with an MGI of 65, 63, 63, and 62%; and MP, of 68, 62, 64, and 63% at 28, 70, 100, and 121 °C, respectively (Figs. 4a and b). As for the stability tests regarding pH variation, the supernatant presented high stability, without significant changes in activity against all the tested fungi (Fig. 4c). Similar results were found in supernatant stability when exposed to UV 365 nm and visible light (Fig. 4d).

### Colonization of B. velezensis CMRP 4489 on soybean seeds

**Figure 5:**
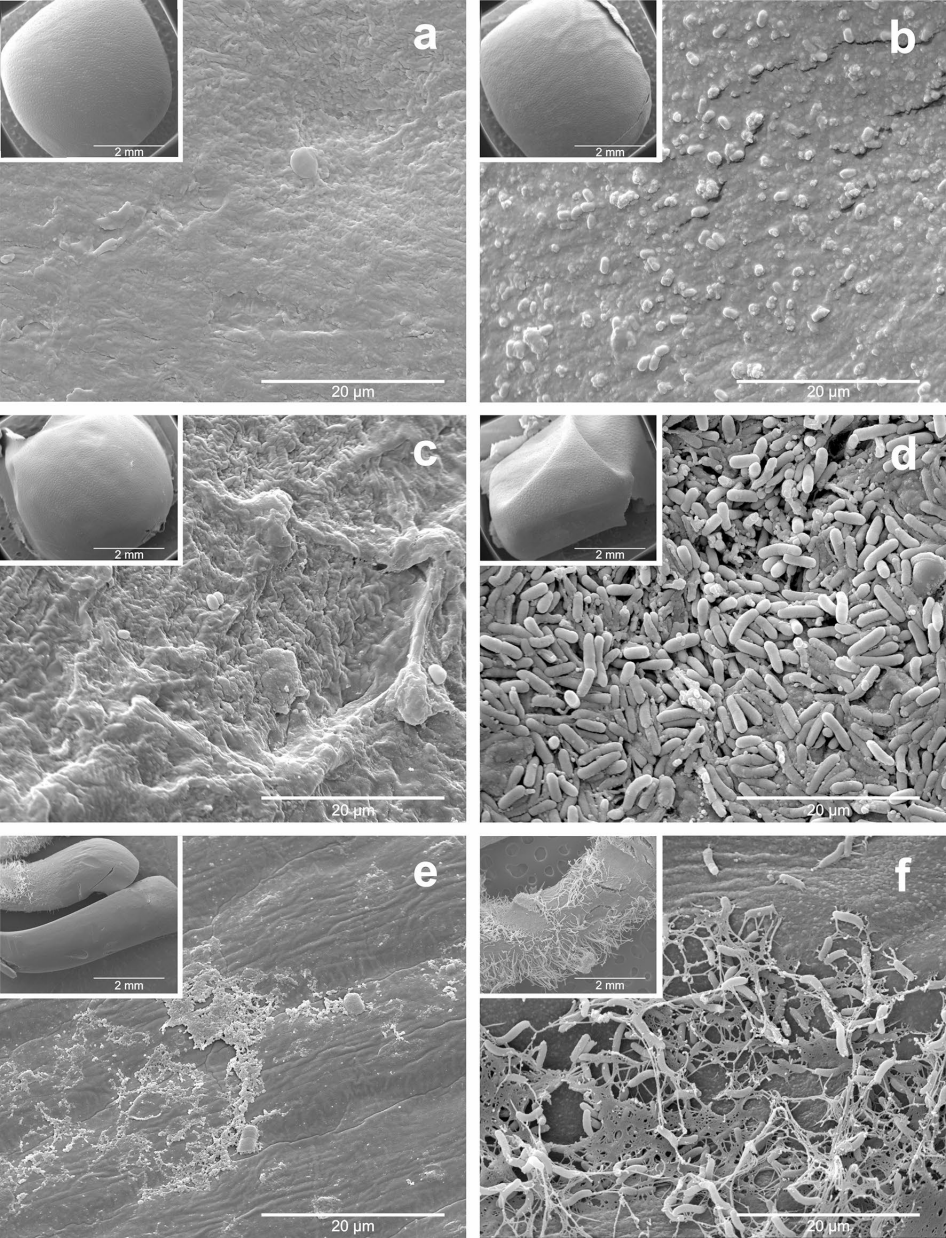
Scanning electron microscopy of the colonization of *Bacillus velezensis* CMRP4489 on soybean seeds. The first column shows untreated seeds. The second shows the seeds treated with strain CMRP4489, adjusted to 5 × 10^9^ CFU/mL (200 mL/100 kg). The first row shows seeds that were fixated on day 0 of treatment; the second row shows the seeds after 7 days of incubation, and the third row shows the radicles of the seeds after 7 days of incubation. All images have inserts at 50 × magnification.

Strain CMRP 4489 was able to attach and multiply on the soybean seed surface. In Fig. 5, the first column shows untreated seeds, while the second indicates the seeds treated with strain CMRP 4489, adjusted to 5 × 10^9^ CFU/mL (200 mL/100 kg). The first row shows seeds that were fixated on day 0 of treatment; the second row shows the seeds after 7 days of incubation, and the third row shows the radicles of the seeds after 7 days of incubation. All images have inserts at 50 × magnification. The control seeds treated with CM2 on day 0 exhibited an absence of adhered microorganisms on the surface (Fig. 5a). Seeds treated with CMRP 4489 on day 0 presented several endospores attached to their surface (Fig. 5b). After 7 days of incubation, the control seeds (Fig. 5c) and radicles (Fig. 5e) were not colonized with any type of microorganism. However, those treated with strain CMRP 4489 after 7 days of incubation showed surfaces fully colonized with a net-shaped biofilm (Fig. 5d). In the radicles of the seeds treated with strain CMRP 4489, after 7 days of incubation, significant colonization was observed, with net-shaped biofilm and exopolysaccharides (Fig. 5f). The quantification results showed that on day 0, the concentration of CMRP 4489 on the soybean seeds was 1.16 × 10^5^ CFU/mL and, after 7 days of germination, its concentration on the seeds was 3.18 × 10^7^ CFU/mL, while the concentration on the radicles was 3.08 × 10^6^ CFU/mL. These results evidence the high potential of CMRP 4489 to colonize soybean roots. The strain transitioned from the seeds to nearby structures, being the first bacterial colonizer of the root system and potentially protective against fungal soil diseases.

### Control of Sclerotinia sclerotiorum on soybean seeds by inoculation of B. velezensis CMRP 4489

**Figure 6:**
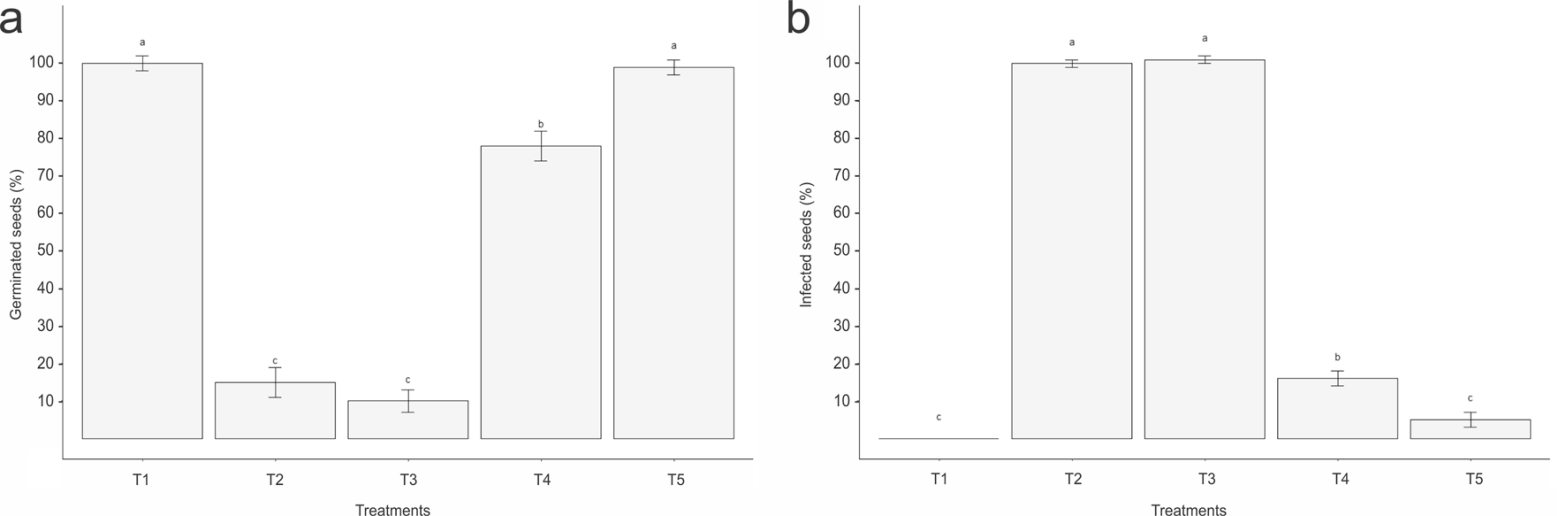
Control of *Sclerotinia sclerotiorum* on soybean seeds by inoculation of *Bacillus velezensis* CMRP 4489. Evaluation of the % of (**a**) germinated seeds and (**b**) uninfected seeds of *Sclerotinia sclerotiorum*. Bars represent the standard deviation based on 13 replicates per treatment. Letters indicate statistical difference by the Tukey test (*p* < 0.05).

In the evaluation of SS suppression in the in vitro infected soybean seeds, the results evidenced the high control potential of the CMRP 4489 strain, in addition to not presenting a negative influence on germination. The tested prototype was prepared with 5 × 10^9^ CFU/mL and antifungal extracellular metabolites produced during the submerged liquid fermentation stage (Fig. 6). The uninfected and untreated control seeds (T1) showed a germination rate of approximately 100% (Fig. 6a), and none of them exhibited SS colonization (Fig. 6b). Meanwhile, the untreated control seeds infected with SS (T2) presented a germination rate of almost 15% (Fig. 6a); however, approximately 95% of the seeds died since they were fully colonized by SS (Fig. 6b). The control seeds infected with the fungus and treated only with sterilized CM2 (T3) showed no significant differences compared to T2 (Figs. 6a and b). On the other hand, the soybean seeds treated with the commercial product (T4) composed of 52.50 g/L of fluazinam and 350.00 g/L of thiophanate-methyl had a germination rate of roughly 80% (Fig. 6a), and the chemical compound suppressed the development of SS. Treatment T4 revealed only around 16% of seeds killed by SS colonization (Fig. 6b). The results showed significant differences when comparing the treated groups to the controls. However, the soybean seeds treated with strain CMRP 4489 presented a germination rate of approximately 95% (Fig. 6a), differing significantly from the controls and the chemical treatment (T4). When evaluating the potential to suppress the development of SS in the in vitro infected soybean seeds, the CMRP 4489 strain (T5) showed high control performance, differing significantly from the controls and the chemical treatment (Fig. 6b). Strain CMRP 4489 suppressed around 95% of SS colonization. This treatment showed that only approximately 5% of the seeds were killed by SS colonization (Fig. 6b). These results are directly related to the extracellular antifungal metabolites produced by the CMRP 4489 strain during submerged liquid fermentation and that were present in the prototype used in the assay, corroborating that, in fermentation processes used specifically to produce antimicrobial metabolites, it is possible to elaborate biological products for seed treatment with high potential for controlling this soil-borne fungal pathogen. Furthermore, due to the high colonization potential of the CMRP 4489 strain, as observed in Subheading 3.8, it probably also contributes to the control of SS.

### Genome mining: prediction of secondary metabolite clusters and comparative analysis of genes involved in biofilm formation

**Figure 7:**
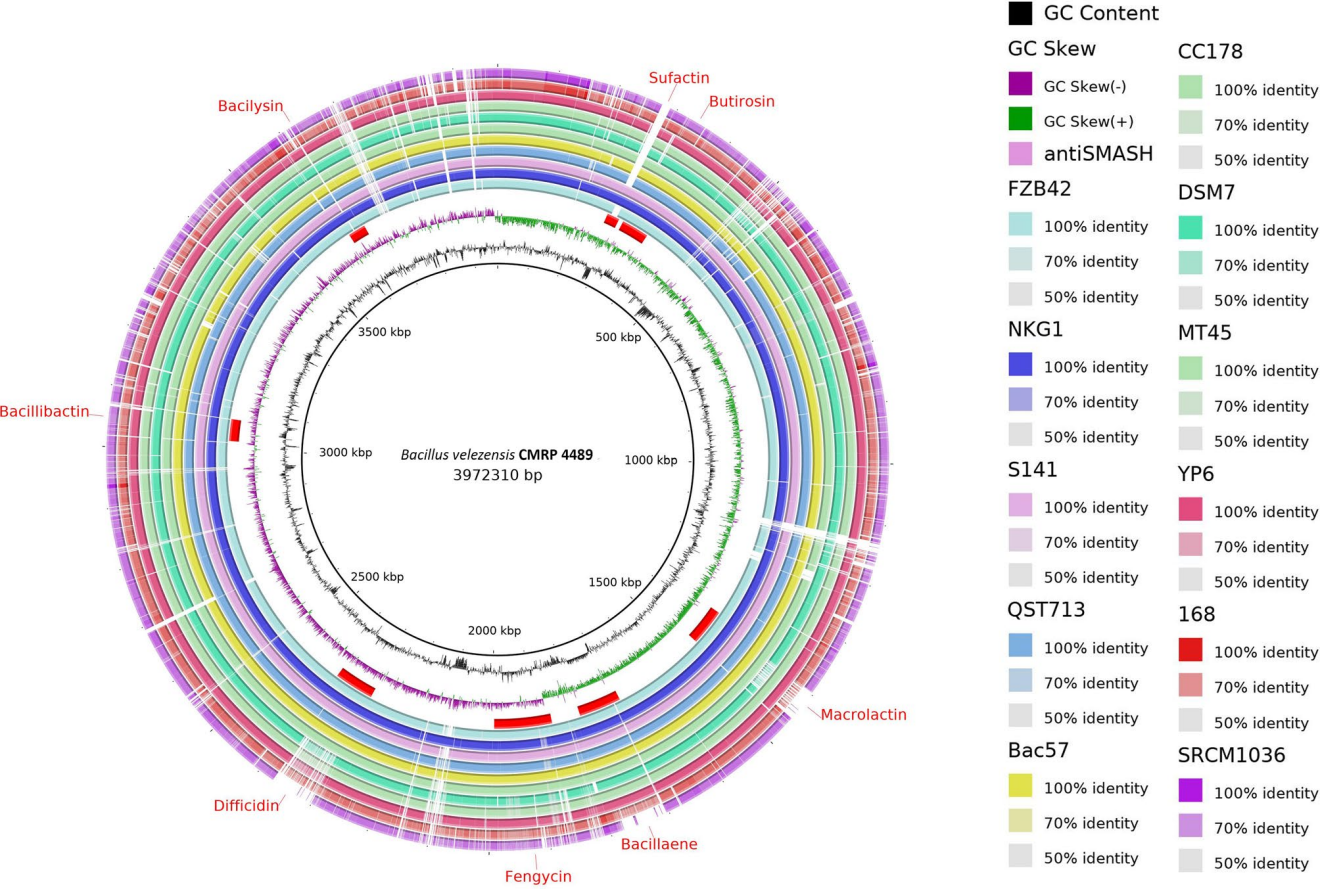
Circular representation and comparison of the *Bacillus velezensis* CMRP 4489 strain genome with other strains from the *Bacillus subtilis* group using the BRIG program. From the inside out: representation of the genome size of *B. velezensis* CMRP 4489, GC Skew, GC Content, positions of the clusters of the *B. velezensis* CMRP4489 strain, and the strains FZB42, NGK1, S141, QST713, Bac57, CC178, DSM7, MT45, YP6, 168, and SRCM1036, respectively.

The antiSMASH 6.0 webserver found 12 BGCs in the genome of the *B. velezensis* CMRP 4489 strain, seven of which presented significant similarity with previously identified clusters in the Minimum Information about a Biosynthetic Gene cluster (MIBiG) repository that are involved in the synthesis of surfactin (82% similarity to known clusters by antiSMASH), macrolactin (100%), bacillaene (100%), fengycin (100%), difficidin (100%), bacillibactin (100%), and bacilysin (100%). The similarity between the clusters of the CMRP 4489 strain and the *Bacillus velezensis* FZB42 strain^28–30^ is noteworthy and considered a model for PGPR and biocontrol strains. The clusters were also represented in a circular genome image (Fig. 7) for comparison with other strains of the *Bacillus subtilis* group. Four out of 12 clusters did not show similarity with any of the clusters in the antiSMASH database. Comparative analysis of the 13 secondary metabolite biosynthesis gene clusters was conducted using antiSMASH 6.0 (Supplementary Table 2). Several genes associated with rhizosphere colonization and biofilm production were found in the CMRP 4489 genome (Supplementary Table 3). Key genes related to the root colonization process were found, including: genes regulating the expression of exopolysaccharides *sinR* (80% similarity with the genes used for comparison) and *sinL* (97%); the operon responsible for the production of exopolysaccharides *epsA-O* (~ 99%); operon *sfrAA-AC* (79%, 75%, 87%), related to surfactin synthesis; operon *swrA-C* (84%, 82%, 80%), important for swarming motility and flagella production, and operon *yqxM-sipW-tasA* (~ 99%), associated with the production of biofilm matrix components. Other genes related to root colonization were also found in the genome of the CMRP 4489 strain (Supplementary Table 3).

## Discussion

Pesticide use is the most diffused strategy to avoid crop losses caused by plant diseases. However, some aspects, such as pathogen resistance^31,32^, environmental contamination^33,34^, and damage to human health^35^, caused by the excessive and incorrect use of these chemical defensives, have fostered a growing interest in bio-based products.

The use of microorganisms for biological product development is a promising alternative that has gained significant attention over the past decades. According to Van Lenteren et al.^36^, over 200 microbial strains are currently being used as commercial products in Brazil, Australia, Canada, the European Union, Japan, New Zealand, and the United States of America.

In the present study, *B. velezensis* CMRP 4489 showed potential antagonism against the analyzed phytopathogenic fungi, as observed in the dual-culture assay. However, for use as a bioproduct, it is necessary to identify the best fermentation system for mass production. Thus, cultivation conditions are important factors that must also be considered in the production of secondary metabolites^37^. In addition to bacterial biomass, antimicrobial compounds need to be present in large quantities in the fermented product. Therefore, many studies have been developed to formulate more suitable culture media for secondary metabolite production^38,39^. In this study, we evaluated two culture media for liquid fermentation. Among the basal media studied, the CM2 medium, supplemented with micronutrients, provided more adequate conditions for strain CMRP 4489 to produce antifungal compounds. In a study on the optimization of multiple responses of biomass and antifungal metabolites with *Bacillus subtilis* EA-CB0015, Mosquera et al.^40^ demonstrated the importance of formulating more suitable culture media to produce high concentrations of biomass and antifungal molecules for biotechnological application. In some cases, depending on the microorganism studied, a simple medium can also provide adequate levels of production of these compounds, as observed with the CM1 medium, and as reported by Zouari et al.^41^ and Torres et al.^42^ when using LB and Mueller–Hinton culture media, respectively.

Aside from nutrient sources, microbial growth also influences the synthesis of biomolecules since molecules from secondary metabolism are more expressed in the stationary phase, as observed in the present study and in Mezghanni et al.^43^. When comparing the growth curve and antifungal activity of the CMRP 4489 strain under the culture conditions used in this study, the optimal production time of antifungal metabolites, in which the highest viable biomass of strain CMRP 4489 was observed, was 36 h after inoculation. Similar results were found in Xiong^44^, where two *B. velezensis* strains demonstrated production of antifungal molecules after entering the stationary phase, which increased during fermentation; these molecules were identified as belonging to the Iturin family, a group of lipopeptides whose BGCs were detected in the genome of CMRP 4489.

Considering that CFS-CM2 presented better performance, it was chosen to evaluate the ultrastructural damage caused to SS. The results showed that CFS-CM2 distorted and damaged SS hyphae and that fungal growth was inhibited, corroborating the results obtained by Farzand et al.^45^ and Zhang L and Sun^46^, who analyzed the effect of lipopeptides produced by *Bacillus* species on ultrastructural changes in fungal hyphae. In another study, the shrinkage of *Lasiodiplodia theobromae* hyphae due to antifungal lipopeptides produced by *B. subtilis* B1 was reported, which is also consistent with our findings^47^.

Among the lipopeptides produced by species of the genus *Bacillus* with fungicidal effect is fengycin. In several reports, fengycin has been shown to cause severe damage to fungal hyphae, resulting in their death^48,49^. Considering that in the present study 12 BGCs were identified in the genome of the *B. velezensis* CMRP 4489 strain and that some of them are involved in the synthesis of lipopeptides, including fengycin, this may explain the possible reason behind the effects observed in the hyphae structure of SS herein. However, further studies with the CMRP 4489 strain are underway to identify the antifungal molecules produced by this strain.

Regarding of BGCs found in the *Bacillus* spp. genome, NRPSs and PKSs were the most common^50^ and were also observed in the genome of *B. velezensis* CMRP 4489. These and other BGCs are responsible for the production of several secondary metabolites of biotechnological interest, being characterized according to their chemical composition as peptides (bacteriocins, bacilysin), lipopeptides (surfactin, iturin, fengycin), and polyketides (difficidin, bacillaene, macrolactin), among others^51^.

The CMRP 4489 strain has several clusters that have not yet been identified among those already described, some of which share similarities with bacterial isolates with biotechnological potential, as is the case of bacillibactin, macrolactin, surfactin, and bacilysin, present in the QST713 strain of the commercial product Serenade^®^^52^, and in strain FZB42 of the commercial product RhizoVital^®^^53,54^. This indicates that CMRP 4489 also has significant biotech potential and could be part of the composition of a commercial product in the near future. Among the clusters that are not shared by reference strains are thiopeptides. These peptides, which are found in the genome of several bacteria, synthesized by ribosomes and modified after translation^55^, may be related to biofilm formation and antibacterial^56^ and antifungal^57^ activity, among others. According to Just Baringo et al.^58^, there are more than one hundred families in this cluster.

Other crucial parameters that were considered in this study were related to the stability of the antifungal effect under adverse conditions. These stability attributes are important for the future use of this strain in bioproducts for plant disease control. The results regarding thermostability showed that there was a decrease in activity as the temperature increased; however, the analyzed compounds did not lose their activity. As for pH stability, there was little variation in activity in the tested pH range. Similarly, Yu et al.^59^ also observed stability in the compounds produced by a strain of *Bacillus* sp. However, while evaluating a strain of *Bacillus* sp., Zhao et al.^60^, reported a significant reduction in activity when the compounds were tested regarding temperature and pH. The photostability tests showed that 12-h exposure to visible light and UV 365 nm did not interfere in the antifungal activity of the supernatant. This was analogous to the compounds of *B. amyloliquefaciens* strain CNU114001, which were also stable under UV light exposure for 30 min^61^.

All tests performed herein were essential for the characterization of the supernatants’ antifungal activity, and their effect was evaluated based on morphological changes in SS hyphae, showing the potential of the CMRP 4489 strain as a biological control agent. However, another important factor for use as a biocontrol agent is its ability to be an excellent colonizer. That is why we evaluated the colonization of *B. velezensis* CMRP 4489 on soybean seeds and the genes involved in the formation and regulation of colonization mechanisms.

In order for the colonization and formation of microbial communities to take place in the root structures of a plant, it is necessary that the microorganisms have the ability to move around and form cell clusters around the roots. Several genes directly or indirectly associated with colonization were found in the genome of the CMRP 4489 strain, highlighting the presence of *srfAA-AC*, genes involved in the production of surfactin that reduce the surface tension of the matrix and allow greater mobility, and *swrA-C*, involved in regulating the expression of flagellins and in improving the swarming capacity of the strain, which is one of the main mechanisms of radicular colonization^62^. In this study, swarming was also evaluated and evidenced in the CMRP 4489 strain in the in vitro assay.

Molecular data from in vitro assays regarding biofilm formation and swarming motility suggest that genes associated with biofilm formation and regulation and motility are functional and may confer the capacity of rhizosphere colonization to the strain^63,64^. Quantitative colonization data associated with electron microscopy images demonstrated that the CMRP 4489 strain is able to adhere and multiply on soybean seeds and radicles, a result similar to that observed by Berlanga-Clavero et al.^18^, who studied the ability of a *B. subtilis* strain to colonize melon seeds and radicles. Based on these data, seed treatment is a viable strategy for using this strain, considering the colonization capacity of CMRP 4489 in seeds and radicles after germination and its antagonistic activity against phytopathogenic fungi. Moreover, its beneficial effects on seed germination make the direct seed coating strategy a possible alternative for its use.

## Conclusions

In recent years, the augmented burden on farmers for increased yields has resulted in excess usage of fertilizers and chemical pesticides. The use of chemical fertilizers and antifungal agents to increase production is not sustainable for obvious reasons. The present study suggested the use of the biocontrol agent *B. velezensis* CMRP 4489 as a new alternative for bioformulation for use in scale in bio-based industries. The in vitro and soybean seeds data obtained herein has been complemented with genomic data of *B. velezensis* CMRP 4489, with mapping of secondary metabolite clusters and comparative analysis of genes involved in biofilm formation, indicating that the biological control agent properties of strain CMRP 4489 are genetic and hence stable.

## Supplementary Information

Supplementary Information.

## Data Availability

The genome analyzed during the current study is available in the DDBJ/EMBL/GenBank under accession number CP023748 (BioProject PRJNA412668, BioSample SAMN07722662).
