# Supplementary material for: Antifungal activity and genomic characterization of the biocontrol agent Bacillus velezensis CMRP 4489
Source: Sci Rep. 2022 Oct 18;12:17401. doi: 10.1038/s41598-022-22380-0 (PMC9579199; doi:10.1038/s41598-022-22380-0)
Supplement: Supplementary file 1 — Supplementary Information. [file 41598_2022_22380_MOESM1_ESM.docx]

**Supplementary Information**

**Antifungal activity and genomic characterization of the biocontrol agent *Bacillus velezensis* CMRP 4489**

Julia Pezarini Baptista^1^, Gustavo Manoel Teixeira^1^, Maria Luiza Abreu de Jesus^1^, Rosiana Bertê^1^, Allan Higashi^1^, Mirela Mosela^1^, Daniel Vieira da Silva^1^, João Paulo de Oliveira¹, Danilo Sipoli Sanches^2^, Jacques Duílio Brancher^3^, Maria Isabel Balbi-Peña^4^, Ulisses de Padua Pereira^5^, and Admilton Gonçalves de Oliveira^1,6,*^

^1^Department of Microbiology, Universidade Estadual de Londrina, 86057-970, Londrina, PR, Brazil

^2^Universidade Tecnológica Federal do Paraná, 86300000, Cornélio Procópio, PR, Brazil

^3^Department of Computer Science, Universidade Estadual de Londrina, 86057-970, Londrina, PR, Brazil

^4^Department of Agronomy, Universidade Estadual de Londrina, 86057-970, Londrina, PR, Brazil

^5^Department of Preventive Veterinary Medicine, Universidade Estadual de Londrina, 86057-970, Londrina, PR, Brazil

^6^Laboratory of Electron Microscopy and Microanalysis, Universidade Estadual de Londrina, 86057-970, Londrina, PR, Brazil

*Correspondence: admilton@uel.br; Tel.: +554333715147


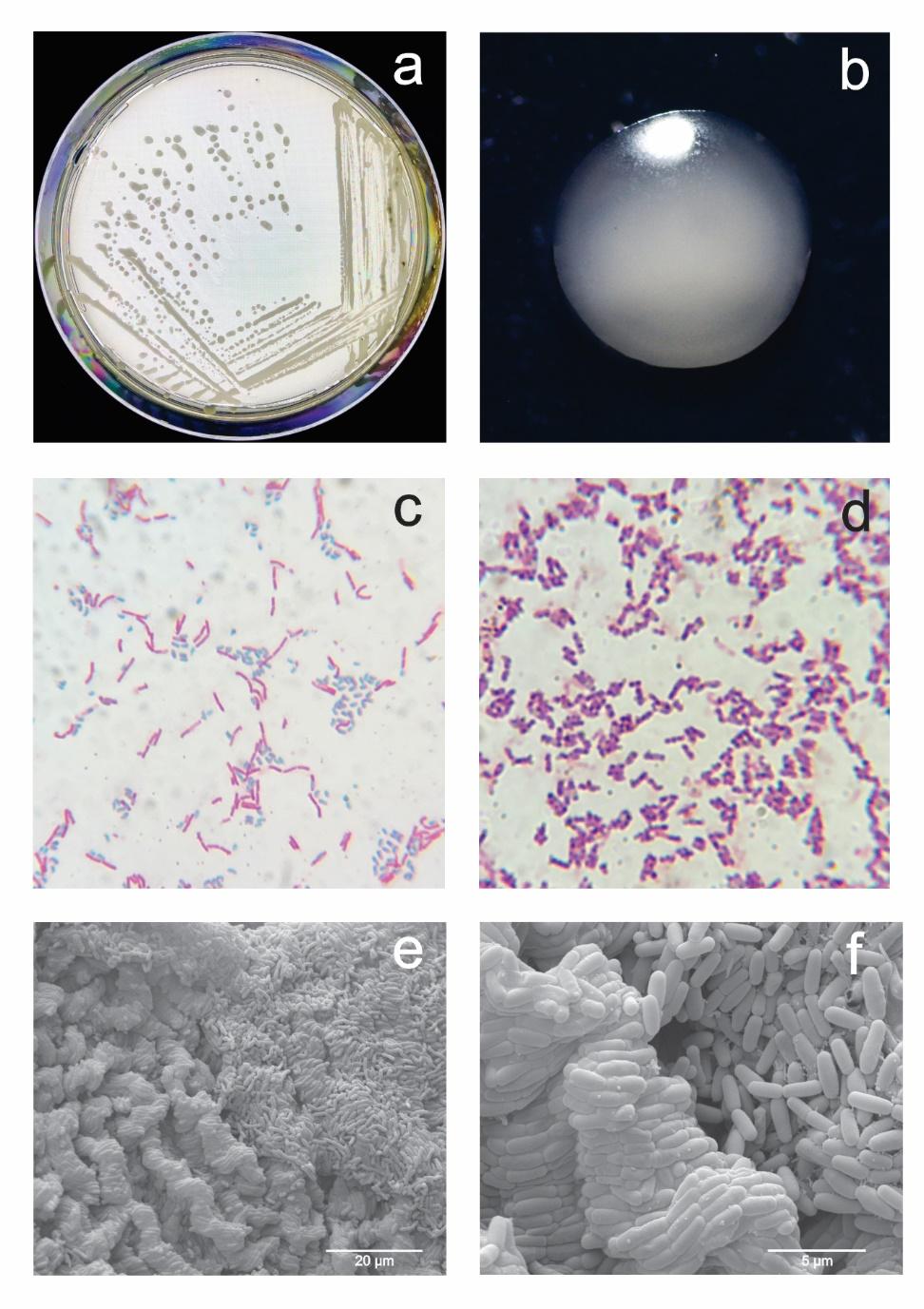


**Supplementary Figure 1:** Phenotypic characterization and colony architecture of *B. velezensis* CMRP4489. **(a)** *B. velezensis* CMRP 4489 grown in Luria-Bertani agar at 28 ºC, 24 h. **(b)** *B. velezensis* CMRP 4489 colony phenotype at 40x magnification; **(c)** Gram staining for morphology and cell wall visualization of *B. velezensis* CMRP 4489; **(d)** Endospore by *B. velezensis* CMRP 4489; **(e)** and **(f)** Scanning electron microscopy at different magnifications of colony of *B. velezensis* CMRP 4489.


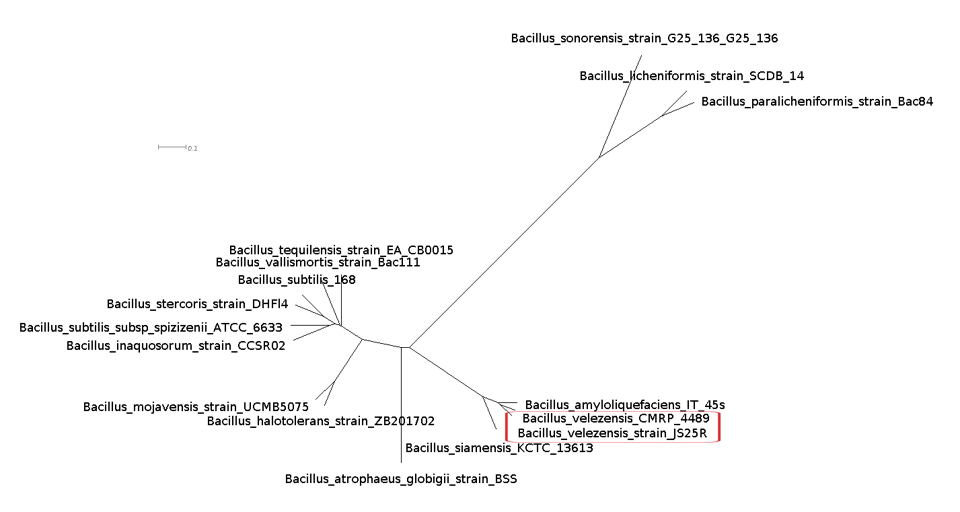


**Supplementary Figure 2:** Phylogenetic tree representing the similarity between the strains of the genus *Bacillus* used and the genome of the isolate CMRP 4489, the genomes highlighted in the red frame refer to the species *Bacillus velezensis*. The matrix used was generated using the Gegenees software and the tree assembled through the SplitsTree.


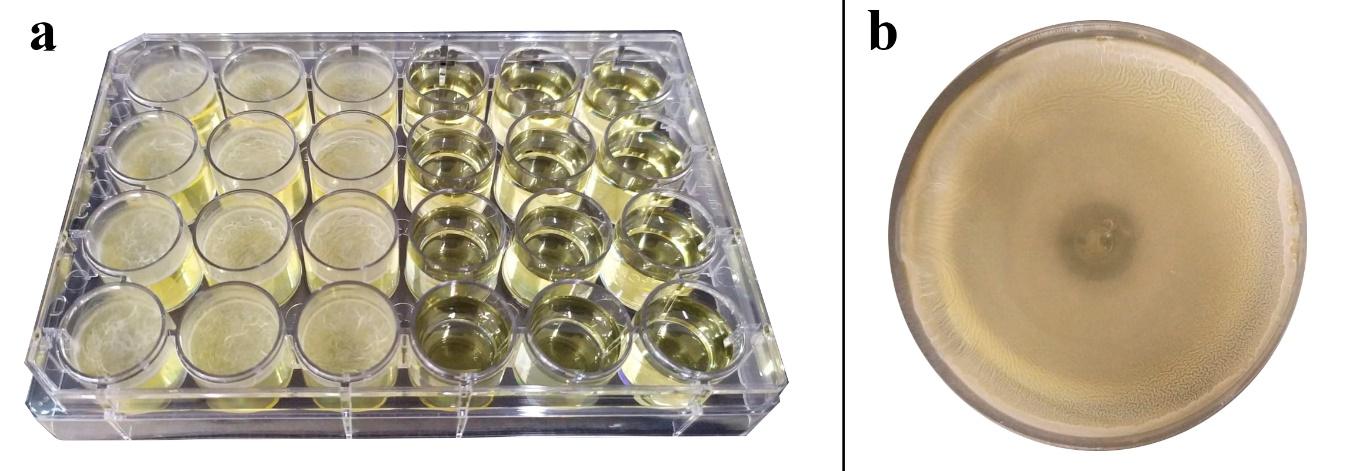


**Supplementary Figure 3:** Images of pellicle formation and swarming motility. **(a)** Pellicle formation on the liquid-air interface in static liquid medium. The first three columns show pellicle formation after the inoculation of *B. velezensis* CMRP 4489; the next three represent the control. **(b)** Complete colonization of a Petri dish after 10μL central inoculation with *B. velezensis* CMRP 4489 in an overnight culture.

**Supplementary Table 1:** ANI and dDDH values of the CMRP 4489 genome compared to the reference genomes of the genus *Bacillus*.

| Reference genome | ANI | DDH |
| --- | --- | --- |
| *Bacillus velezensis* strain JS25R | 98.35 | 85.10 |
| *Bacillus amyloliquefaciens* IT 45s | 97.74 | 80.20 |
| *Bacillus siamensis* KCTC 13613 | 94.32 | 56.70 |
| *Bacillus inaquosorum* strain CCSR02 | 77.59 | 21.10 |
| *Bacillus atrophaeus globigii* strain BSS | 77.34 | 20.90 |
| *Bacillus tequilensis* strain EA CB0015 | 77.28 | 20.90 |
| *Bacillus mojavensis* strain UCMB5075 | 77.27 | 20.70 |
| *Bacillus subtilis* subsp. *spizizenii* ATCC 6633 | 77.25 | 20.80 |
| *Bacillus halotolerans* strain ZB201702 | 77.18 | 20.60 |
| *Bacillus subtilis* 168 | 76.87 | 20.70 |
| *Bacillus vallismortis* strain Bac111 | 76.85 | 20.70 |
| *Bacillus stercoris* strain DHFl4 | 76.71 | 20.50 |
| *Bacillus licheniformis* strain SCDB 14 | 72.73 | 19.40 |
| *Bacillus paralicheniformis* strain Bac84 | 72.59 | 19.80 |
| *Bacillus sonorensis* strain G25 136 G25 136 | 72.51 | 19.20 |

**Supplementary Table 2:** Comparative analysis of secondary metabolite biosynthesis gene clusters using antiSMASH 6.0.

| REGION | TYPE | FROM | TO | SIZE | MOST SIMILAR KNOW CLUSTER | | SIMILARITY |
| --- | --- | --- | --- | --- | --- | --- | --- |
| Region 1 | Thiopeptide,LAP | 266.234 | 295.970 | 29.736 |  |  |  |
| Region 2 | NRPS | 306.792 | 371.874 | 65.082 | *[surfactin](https://mibig.secondarymetabolites.org/go/BGC0000433/1) | NRP:Lipopeptide | 82% |
| Region 3 | PKS-like | 903.192 | 944.436 | 41.244 | *[butirosin A / butirosin B](https://mibig.secondarymetabolites.org/go/BGC0000693/1) | Saccharide | 7% |
| Region 4 | Terpene | 1.030.062 | 1.047.233 | 17.171 |  |  |  |
| Region 5 | transAT-PKS | 1.376.006 | 1.462.392 | 86.386 | *[macrolactin H](https://mibig.secondarymetabolites.org/go/BGC0000181/1) | Polyketide | 100% |
| Region 6 | transAT-PKS, T3PKS, NRPS | 1.688.525 | 1.789.076 | 100.551 | *[bacillaene](https://mibig.secondarymetabolites.org/go/BGC0001089/1) | Polyketide + NRP | 100% |
| Region 7 | NRPS, transAT-PKS, betalactone | 1.857.379 | 1.994.553 | 137.174 | *[fengycin](https://mibig.secondarymetabolites.org/go/BGC0001095/1) | NRP | 100% |
| Region 8 | Terpene | 2.017.590 | 2.039.473 | 21.883 |  |  |  |
| Region 9 | T3PKS | 2.123.150 | 2.164.250 | 41.100 |  |  |  |
| Region 10 | transAT-PKS | 2.296.150 | 2.389.942 | 93.792 | *[difficidin](https://mibig.secondarymetabolites.org/go/BGC0000176/1) | Polyketide + NRP | 100% |
| Region 11 | NRPS, RiPP-like | 3.022.525 | 3.074.316 | 51.791 | *[bacillibactin](https://mibig.secondarymetabolites.org/go/BGC0000309/1) | NRP | 100% |
| Region 12 | other | 3.602.828 | 3.644.246 | 41.418 | *[bacilysin](https://mibig.secondarymetabolites.org/go/BGC0001184/1) | Other | 100% |

* Hyperlinked with repository MIBiG referring to the respective cluster.

**Supplementary Table 3:** Genes related to biofilm formation/regulation in *Bacillus velezensis* CMRP 4489.

| Genes | Genome position in *Bacillus velezensis* CRMP 4489 (bp) | | | Identity with reference (%) | Described function according to Subtwiki (48) |
| --- | --- | --- | --- | --- | --- |
| *abrB* | 31511 | - | 31222 | 91.034 | Transcriptional regulator of transition state genes |
| *degQ* | 3020158 | - | 3020023 | 86.765 | Stimulates production of degradative enzymes and extracellular poly-gamma-glutamate; stimulates phosphorylation of DegU by DegS; gene is not expressed in lab strain 168 due to promoter down-mutation in the -10 region (T - 10 ---> C) |
| *degU* | 3403559 | - | 3402870 | 86.377 | Two-component response regulator; regulation of degradative enzyme expression, genetic competence, biofilm formation, capsule biosynthesis (together with SwrA); non-phosphorylated DegU is required for swarming motility |
| *epsA* | 3306148 | - | 3305441 | 98.87 | Extracellular polysaccharide synthesis; putative transmembrane modulator of EpsB activity; might activate EpsB autophosphorylation and substrate phosphorylation |
| *epsB* | 3305435 | - | 3304755 | 99.853 | Extracellular polysaccharide synthesis; protein tyrosine kinase; phosphorylation of EpsE |
| *epsC* | 3304509 | - | 3302716 | 99.108 | UDP-N-acetylglucosamine 4,6-dehydratase; required for extracellular polysaccharide synthesis; this gene is inactive in *B. subtilis* 168 |
| *epsD* | 3302700 | - | 3301561 | 98.333 | Extracellular polysaccharide synthesis |
| *epsE* | 3301564 | - | 3300722 | 99.288 | Inhibitor of motility and glycosyltransferase required for EPS biosynthesis |
| *epsF* | 3300729 | - | 3299593 | 99.033 | Similar to glycosyltransferase |
| *epsG* | 3299589 | - | 3298486 | 99.275 | Extracellular polysaccharide synthesis |
| *epsH* | 3298467 | - | 3297430 | 98.94 | Undecaprenyl (UnDP) priming UDP-N-acetyl-glucosamine transferase; synthesis of extracellular poly-N-acetylglucosamine |
| *epsI* | 3297425 | - | 3296349 | 98.514 | Glycosyltransferase; synthesis of extracellular poly-N-acetylglucosamine |
| *epsJ* | 3296352 | - | 3295318 | 98.647 | UDP-N-acetyl-glucosamine transferase; synthesis of extracellular poly-N-acetylglucosamine |
| *epsK* | 3295321 | - | 3293804 | 98.88 | Export of extracellular poly-N-acetylglucosamine |
| *epsL* | 3293807 | - | 3293199 | 99.507 | Similar to UDP-galactose phosphate transferase; extracellular polysaccharide synthesis |
| *epsM* | 3293202 | - | 3292555 | 99.074 | UDP-2,4,6-trideoxy-2-acetamido-4-amino glucose acetyltransferase; extracellular polysaccharide synthesis |
| *epsN* | 3292550 | - | 3291378 | 98.892 | UDP-2,6-dideoxy 2-acetamido 4-keto glucose aminotransferase; required for extracellular polysaccharide synthesis |
| *epsO* | 3291399 | - | 3290434 | 99.275 | Similar to pyruvyltransferase; extracellular polysaccharide synthesis |
| *galE* | 1166735 | - | 1165743 | 99.799 | UDP glucose 4-epimerase |
| *kinA* | 1344665 | - | 1346485 | 77.669 | Two-component sensor kinase; phosphorylates Spo0F; part of the phosphorelay |
| *motA* | 1311733 | - | 1310918 | 79.044 | H+-coupled MotA-MotB flagellar stator |
| *motB* | 1310946 | - | 1310204 | 76.573 | H+-coupled MotA-MotB flagellar stator |
| *remA* | 1560848 | - | 1561117 | 97.407 | Transcriptional regulator of the extracellular matrix genes; acts in parallel with SinR, AbrB, and DegU |
| *sigD* | 1635695 | - | 1636459 | 87.059 | RNA polymerase sigma factor SigD |
| *sigH* | 97065 | - | 97702 | 87.774 | RNA polymerase sigma factor SigH; not fully active in laboratory strains due to a mutation (V117A) |
| *sigW* | 174099 | - | 174662 | 82.979 | RNA polymerase ECF-type sigma factor SigW; required for the adaptation to membrane active agents; activated by alkaline shock and polymyxin B, vancomycin, cephalosporin C, D-cycloserine, and triton X-100 |
| *sinl* | 2449512 | - | 2449685 | 79.661 | Antagonist of SinR; drives SlrR from the SlrR(LOW) to the SlrR(HIGH) state |
| *sinR* | 2449719 | - | 2450054 | 97.024 | Transcriptional regulator (Xre family) of post-exponential-phase responses genes |
| *sipW* | 2451536 | - | 2450952 | 99.145 | Bifunctional signal peptidase I that controls surface-adhered biofilm formation and processes TasA and TapA |
| *spo0A* | 2416347 | - | 2415547 | 99.126 | Phosphorelay regulator; initiation of sporulation; coordinates DNA replication and initiation of sporulation by binding to sites close to the oriC |
| *srfAA* | 345010 | - | 347021 | 79.3 | Surfactin synthetase / competence |
| *srfAB* | 337462 | - | 348217 | 74.887 | Surfactin synthetase / competence |
| *srfAC* | 348257 | - | 352053 | 87.02 | Surfactin synthetase / competence |
| *swrA* | 3378990 | - | 3378654 | 84.366 | Master activator of flagellar biosynthesis; modulator of DegU activity; converts DegU-P from a repressor to an activator of the fla-che operon; enhances sigD transcription; controls the number of flagellar basal bodies; inactive pseudogene in strain 168 |
| *swrB* | 1636487 | - | 1636665 | 81.564 | Control of SigD activity; required for full SigD activity; activates the flagellar type-III secretion export apparatus by the membrane protein FliP |
| *swrC* | 665630 | - | 668773 | 80.17 | Similar to acriflavin resistance protein |
| *tasA* | 2450887 | - | 2450102 | 99.491 | Major component of the biofilm matrix; forms amyloid fibers |
| *yhxB* | 897905 | - | 899647 | 99.369 | Alpha-phosphoglucomutase; required for UDP-glucose synthesis; inhibits FtsZ ring assembly (indirect effect due to a defect in UDP-glucose synthesis); possesses secondary phosphoglucosamine mutase activity |
| *yqxM* | 2452179 | - | 2451508 | 98.958 | TasA anchoring/assembly protein |
